# Effects of virtual reality versus motor imagery versus routine physical therapy in patients with parkinson’s disease: a randomized controlled trial

**DOI:** 10.1186/s12877-024-04845-1

**Published:** 2024-03-05

**Authors:** Muhammad Kashif, Abdulaziz Aoudh Albalwi, Ayesha Zulfiqar, Kiran Bashir, Ahmad Abdullah Alharbi, Shiza Zaidi

**Affiliations:** 1 Riphah College of Rehabilitation and Allied Health Sciences, Riphah International University, Islamabad, Pakistan; 2 Department of Health Rehabilitation Sciences, Faculty of Applied Medical Sciences, University of Tabuk, Tabuk, Saudi Arabia; 3 Margalla Institute of Health Sciences, Islamabad, Pakistan

**Keywords:** Parkinson’s disease, Motor function, Balance, Virtual reality, Motor imagery, Routine physical therapy

## Abstract

**Background:**

Parkinson’s Disease (PD) is the second most common progressive neurodegenerative disorder, mostly affecting balance and motor function caused mainly by a lack of dopamine in the brain. The use of virtual reality (VR) and motor imagery (MI) is emerging as an effective method of rehabilitation for people with Parkinson’s disease. Motor imagery and virtual reality have not been compared in patients with Parkinson’s disease. This randomized clinical trial is unique to compare the effects of virtual reality with routine physical therapy, motor imagery with routine physical therapy, and routine physical therapy alone on balance, motor function, and activities of daily living in patients with Parkinson’s disease.

**Methods:**

A total of sixty patients with Parkinson’s disease were randomized into three groups using lottery method; twenty with virtual reality therapy in addition to physical therapy (group A = VR + RPT), twenty with imagery therapy in addition to physical therapy (group B = MI + RPT), and twenty were treated with only routine physical therapy (group C = RPT). All patients were evaluated using the Unified Parkinson’s Disease Rating Scale (UPDRS) for motor function and activities of daily living, the Berg balance scale (BBS) for balance, and the Activities-specific Balance Confidence Scale (ABCs) for balance confidence at baseline, six and twelve weeks, and one month after treatment discontinuation. The one-way ANOVA was used to compare the outcomes between three groups, and the repeated measures ANOVA was used to compare the outcomes within each of the three groups at a significance level of p-value = 0.05.

**Results:**

According to UPDRS III, the VR + RPT group showed significant improvement in motor function, compared to the MI + RPT and RPT groups, as the Mean ± SD at baseline was 33.95 ± 3.501 and at the 12-week assessment was 17.20 ± 9.451 with a p-value = 0.001. In the VR + RPT group, the BBS score at baseline was 37.15 ± 3.437 and at 12th week was 50.10 ± 4.897 with a p-value = 0.019. Among the VR + RPT group, the ABCS score showed significant improvement as the M ± SD at baseline was 57.95 ± 4.629, and at the 12th week was 78.59 ± 6.386 with a p-value = 0.010. At baseline, the UPDRS II for activities of daily living in the VR + RPT group was 25.20 ± 3.036 and at 12th week it was 15.30 ± 2.364 with p-value of 0.000.

**Conclusion:**

The current study found that the combination of VR and RPT proved to be the most effective treatment method for improving balance, motor function, and activities of daily living in patients with Parkinson’s disease when compared to MI + RPT or RPT alone.

## Introduction

Parkinson’s disease (PD) is reported as the second most commonly prevalent neurodegenerative disorder, chronic in nature, manifested by bradykinesia, rigidity, tremors in resting position and postural instability along with other motor and non-motor symptoms [1]. Slowly developing disease involves nearly one million Americans and 10 million individuals globally [2, 3]. The major etiological factors include age and genetic predisposition. The risk ratio varies between different ethnic backgrounds owing to the contributions by the genetic background and the environmental factors [4]. Many of the studies reported in literature have described male gender dominance for the disease [5]. Prevalence of PD among the general population is reported to be 0.3% while looking at the elderly population; the rate is 1–2% worldwide [6]. Physical rehabilitation is aimed at making the patients independent in their activities of daily living using the assistive strategies or compensatory strategies, enhancing the motor skills using the basic concepts of motor learning and control [7, 8]. Non-pharmacological treatment protocols have been used to increase strength, physical functioning, enhancing balance and improving the gait speed in patients with PD [8, 9]. Stretching exercises, progressive resistance training, aerobics, relaxation strategies, balance and strengthening, to name a few have been studied previously [9]. However, many of these studies incorporating physical therapy regimes have described loss of the effects after the discontinuation of the treatment protocols. Moreover, many barriers to the exercise compliance have been reported in patients with PD. These include treatments required for longer durations; financial constraints are few of the barriers reported [10, 11].

The motor learning concept is the right explanation to the use of virtual reality (VR) for patients with PD Moreover, this technology is useful in maintaining the user’s interest in the treatment protocol for a longer duration of time [12]. Therefore, VR is considered an important adjunct therapy to the traditional physical therapy protocols for patients with PD. Despite the benefits of using VR in rehabilitation, many of the VR based equipment are not easily accessible with major focus on high-end gear or software and neglecting the feasibility to use in different settings [13]. The solution is sought by promoting the use of consumer grade technology to benefit from VR in its true sense. In this perspective, Nintendo Wii gaming system has been a choice among the VR based gaming systems in clinical as well as academic settings [13]. VR system enhance the motor re-learning that in turn leads for neural plasticity, improving the brain functioning and enhancing the physical functioning among the elderly [14]. This theme is also used in the treatment protocols of patients with traumatic brain injury, vestibular dysfunctions, PD, cerebrovascular accidents and cerebral palsied children [15–19].

Moreover, boosting the attention span, enhancing the feelings of achievement, self-esteem and motivation levels among the patients with different neurological dysfunctions is another trademark of VR technology. Reward system motivates the patient during the game time. This also leads to secretion of dopamine from the basal ganglia striatum [20]. This enhanced secretion is thought responsible for the betterment seen in the patients’ performance and learning and acquiring the skills. To conclude, use of VR during the rehabilitation leads to enhanced performance in lieu of the enhanced environmental experiences during the game play [21].

A cognition-based technique, Motor Imagery (MI), is another link used for the rehabilitation of patients with PD. This technique requires paying attention to sequential patterns of learned activities either visually of kinesthetically. The patients with PD are known to have intact locomotor imagery abilities during the on-medication phase despite the fact that the supplementary motor areas not properly functional due to the indirect influence of basal ganglia. Evidence exists about the activation of alternate brain areas through the MI though the left parietal cortex is known to play the responsibility of planning the motor activities [22]. It is believed that during the MI the primary and secondary motor areas (all areas known to be involved in the planning and execution of the motor tasks) are active [23]. Using MI in the rehabilitation protocol of patients with PD has been reported in the literature with success in variety of neurological manifestations [24]. The treatment plans that are task oriented, customized made, goal based, repetitive, regularly performed, exciting, engaging the patients and based on feedbacks are few of the salient feature of the VR based therapy [25, 26].

Strong evidence exists that learning occurs under the roof of explicit and implicit processes. These processes are embedded in VR and MI respectively. Moreover, MI has also been known to augment the effects of VR, therefore promoting and consolidating learning process. Multiple sensory receptors have been thought to be activated by these techniques [27]. In a study of stroke patients, motor imagery and virtual reality were found to increase motor function. Virtual reality, however, was not found to be superior to motor imagery in this study [28]. In recent studies, VR and MI were combined for PD patients to improve balance, motor function, and activities of daily living [29, 30]. To the best of the author’s knowledge, no studies have been done comparing VR with MI in PD patients. Therefore, further research is needed to compare the VR with MI and determine the most effective technique for treating patients with PD. Thus, considering that MI and VR applications are increasingly emerging as potentially useful techniques for rehabilitation in PD, the current study aims to compare the effects of virtual reality with physical therapy, motor imagery with physical therapy, and physical therapy alone on patients with Parkinson’s disease.

## Methods

### Study design

The two-arm, parallel-design clinical trial took place in 2021 at the Department of Physical Therapy at the Safi Hospital in Faisalabad, Pakistan. A single blinded study was conducted in which the assessor was the only one blinded. Patients and the principal investigator were not blinded because of the nature of the intervention. By providing the data in anonymized form and precoding it before handing it over, the statistician was also kept blinded from the group allocation.

### Study participants

An experienced neurologist diagnosed the subjects with PD according to Gelb’s criteria [31]. PD patients were recruited from neurology and neurosurgical departments of tertiary care Hospitals in Faisalabad. After being referred to the Department of Physical Therapy, Safi Hospital, the patients were further evaluated by the physical therapist (a movement specialist) to determine their eligibility to participate in this study according to the inclusion and exclusion. Study participants were 50 to 80 years old with idiopathic Parkinson’s disease, a severity ranging from stage I to stage III on a modified H and Y scale, and intact cognition (a score greater than 24 on the mini-mental score examination (MMSE) [31] as well as transfer independence. A history of Parkinson’s disease surgery, virtual games being used for treatment in the last three months, and virtual game phobia were excluded from the study. Patients with any other neurological presentation, orthopedic pathology, visual anomalies, cardiovascular problems, severe dyskinesia or “on–off” phases were also excluded. Before participating in the study, participants signed an informed consent form.

### Sample size calculation

The study sample size was calculated using the mean Unified Parkinson’s Disease Rating Scale (UPDRS) as 25.1 ± 12.8 and 18.5 ± 11.0 for the VR group and control group, respectively, with a confidence interval (α) of 95% and 80% power of the study extracted from Yang et al. [32]. To detect statistically significant differences, 57 patients were required.

### Randomization

**Fig. 1:**
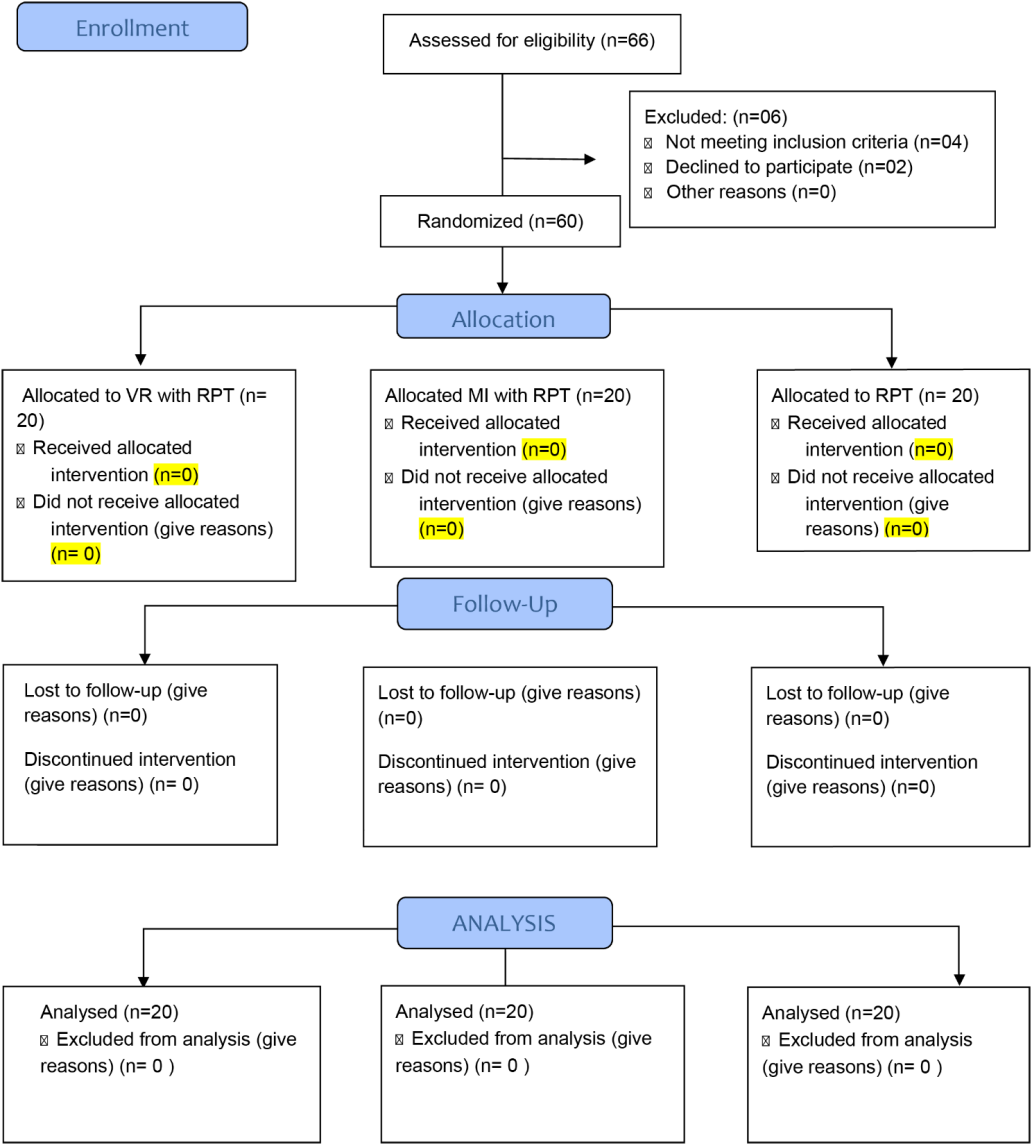
Consort flow sheet of the RCT

In order to carry out randomization following baseline assessment, a lottery procedure was used. For the minimization process, demographic variables were used as inputs. The main auditor assigned each participant a number, and the numbers were then drawn randomly from a box. In this study, the 1:1:1 ratio was maintained for the VR + RPT, MI + RPT, and RPT alone groups. This study’s CONSORT diagram is shown in Fig. 1.

### Groups and intervention procedures

After completing the informed consent form, the subjects volunteered to participate in the study, and were then randomly divided into three groups. Each group consisted of 20 participants at baseline. VR with routine PT were administered to Group A, MI with routine PT were administered to Group B, and Routine PT alone was administered to Group C. All three groups received a 40-minute routine physical therapy per session. The subjects were required to attend 36 interventions during study period. The minimum attendance rate for enrollment of this study was at least 33 sessions attended by each participant.

Study objectives and group allocation were unknown to the independent assessor evaluating the subjects. UPDR-III for motor function, BBS for balance, ABCs for balance confidence, and UPDRS-II for ADLs were used to assess at baseline, 6th, 12th and 16th week. The intervention and assessments took place at the same time of day and in the ON medication state (2 hours after taking the medication) [33, 34]. Patients were assessed late in their ON phase because of the pharmacodynamics of levodopa (the onset of medication effect is 20–40 minutes and the duration of effect is 2–4 hours after medication) [35, 36]. Furthermore, the medication regimens of all study participants remained unchanged throughout the period of the study. Because of the potential of interference in the results of the study, patients with on-off motor fluctuation and dyskinesia above grade 3 on the UPDRS were excluded from the study [37]. Participants did not report any changes in medications during the trial, so no participant was dropped out due to medication changes.

### Interventions

Three groups were randomized to receive VR with routine PT and MI with routine PT and Routine PT only based on a previously described protocol for PD rehabilitation [33].

### Group A: virtual reality and routine PT (VR + RPT)

Group-A participants received three 60-minute sessions per week (which included VR training for 15 to 20 min and routine PT for 40 min) for 12 weeks. The routine PT for 40 min is the same as for Group C.

### VR rehabilitation protocol

**Fig. 2:**
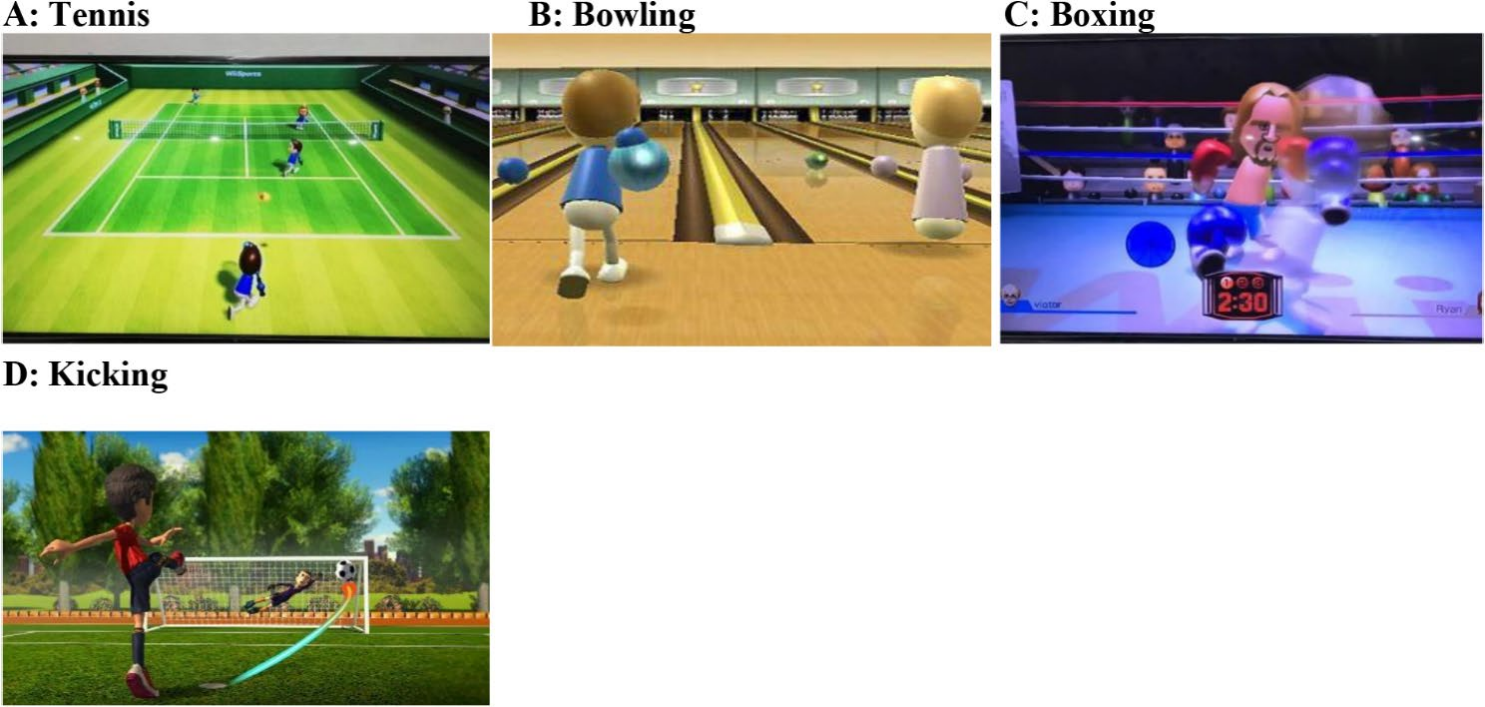
Games for motor function in VR training for PD patients

**Fig. 3:**
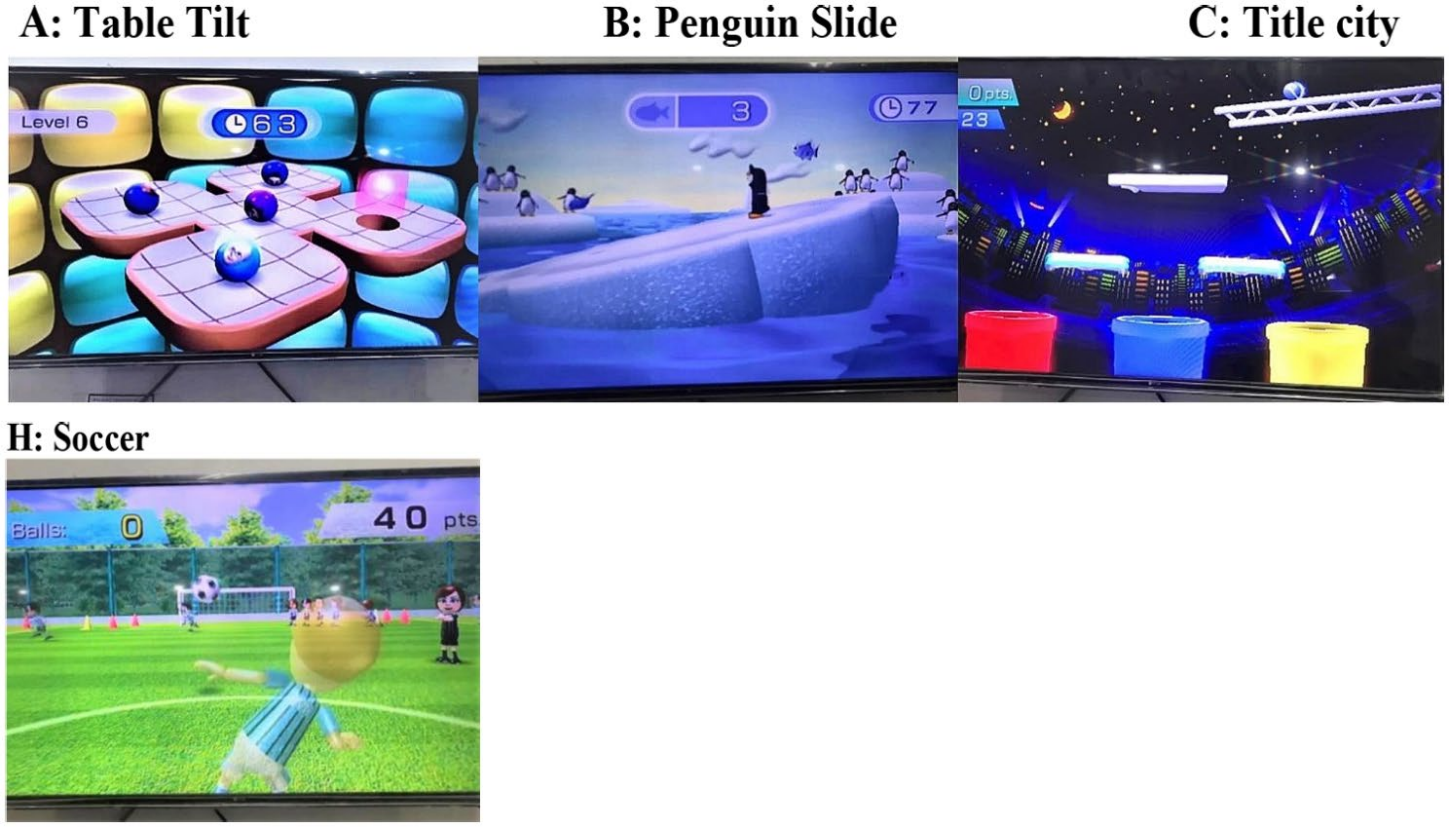
Games used for dynamic balance in VR training for PD patients

**Fig. 4:**
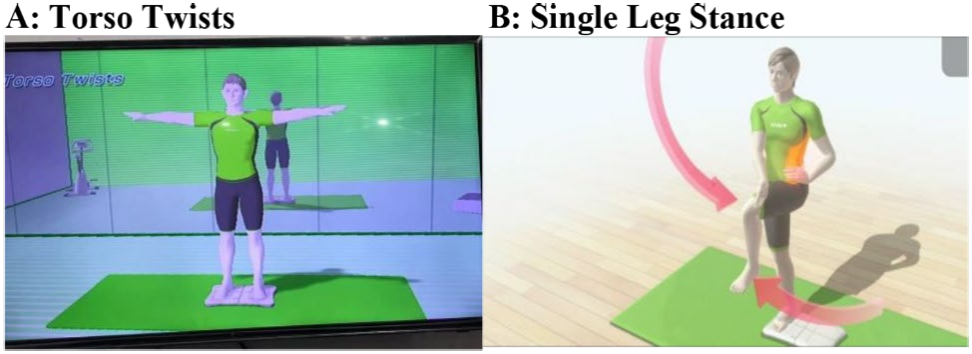
Games used for static balance in VR training for PD patients

VR sessions lasted between 15 and 20 min for each participant. Virtual reality systems are comprised of Wii boxes, Wii controllers, and Wii Fit boards. Patients were advised to play games on the Wii Fit board and engage with the VR system. games for three domains: motor functioning, balance, and ADL Three senior physical therapists (movement experts) selected games for motor functioning, balance, and ADLs based on a prior systematic study [34]. To familiarize the participants with the VR system and the setting, two rehearsals were provided. A description of the games, the treatment, and the score was given to the patients. During the first three weeks, the games were simple. Among the games used to improve motor function were tennis, boxing, bowling, and kicking (Fig. 2), while soccer, table tilt, penguin slide, and tilt city were used to enhance dynamic balance and single-leg extension, and torso twisting were used to improve static balance [29, 35–37].. (Fig. 3 and 4)

With their shoes off, the patients stood within parallel bars on the Wii Fit board. In addition to constant supervision and guidance, the therapist provided timely feedback to the patients. In the VR session, balancing games were played first. At each training session, dynamic balance games and static balance games were played. Depending on the patients’ results, the degree of difficulty of the exercises was progressively raised. After starting with the penguin slide, the patients moved on to table tilt, tilt city, and then soccer. Initially, each game was played for 2–3 min. With each improvement in performance, three to four minutes of table tilting were added. Weight shifts and movement patterns improved after playing this video game. Single leg extensions were performed for up to two minutes per day by the patients. Following the first week, soccer, torso twists, and a tilt city exercise were introduced. These tasks were completed between one and five minutes per session for each individual. After passing the bowling and tennis lessons, a variety of motor function sports were taught, including kicking, boxing, and tennis (the latter being the most challenging). The majority of games can be completed without assistance. Due to an increase in balance and coordination requirements, boxing was used in the last 3 weeks of treatment [33]..

### Group B: motor imagery and routine PT (MI + RPT)

Group-B participants received three 60-minute sessions per week (which included MI training for 15 to 20 min and routine PT for 40 min) for 12 weeks. The routine PT for 40 min is the same as for Group C.

### MI rehabilitation protocol

The MI was implemented in three steps during the last 5–10 min of the session. The first step was for participants to view the recorded footage. Two sets of videos were shown: one with normal motions and another with patients performing the moves. The patients compared and contrasted the two movies. They were then instructed to calm their breathing patterns and relax. In order to sit comfortably and relax on a chair, participants were instructed to have their arms and back supported. With their eyes closed, they concentrate on slow nasal breaths while closing their eyelids. A total of ten times were performed. Following that, verbal instructions were given to the individuals to complete the tasks. As a result of recall, abnormal movement components were highlighted.

### Group C: routine PT (RPT)

Group-C participants received three 60-minute sessions per week (which included routine PT for 40 min and 20-minute walking and cycling with rest periods) for 12 weeks.

### Routine PT treatment protocol

First, patients were given warm-up exercises. The patients inhaled and exhaled while sitting in a chair with their backs and feet supported. In total, five repetitions of each exercise were performed for five minutes to warm up. Patients were taught correct breathing techniques so as to minimize shallow breathing, pushing, and holding their breaths. Supine on the bed, they were instructed to rehearse under the primary investigator’s watchful eye. Stretching was performed four times within 15 min for each area: upper chest and neck flexors, shoulder and adductor muscles, elbow and wrist flexions, knee flexions, calves, and lower back. Strength training sessions lasted 15 min, with each exercise repeated 10–15 times. The primary focus of this workout was on core muscles (abdominals) and hip, knee, back, and elbow extensors. The shoulders flexors, adductors, hip flexors, and knee flexors were stretched for 5 min as a cool-down [33, 38]..

### Adverse event records

An adverse event is any unfavorable medical event that occurs to a patient during or after treatment in a clinical study [32]. Discomfort, nausea, dizziness, and vertigo, are well reported and common negative consequences of VR [43], also known as cyber or simulator sickness [44]. During the trial, all adverse events were considered and no adverse event was reported during the study.

### Outcome measures

At baseline, the 6th, the 12th week of therapy, as well as at the 16th week of follow-up, the motor function of the patient with Parkinson’s was assessed using UPDRS part III by a blind assessor. A renowned self-report and clinical observation tool, UPDRS is routinely used for assessing and monitoring motor function in patients with PD using a variety of paradigms. Subscale III of UPDRS was used in this study for rating motor function. Rigidity, bradykinesia, tremor, and mobility are all scored by the UPDRS. Excellent internal consistency was found in many UPDRS studies [39, 40]. The symptoms can be tracked comprehensively, efficiently, and adaptably with the UPDRS Section III. With a total potential score of 56, each element is graded from 0 to 4. Higher scores indicate more disability [41]. BBS has been reputable as the most commonly used assessment tool in combination with UPDRS, in the clinical settings rehabilitating patients with PD with high inter-rater and intra-rater reliability [42, 43]. ABCs scale is a self-administered scale used as for prediction of fall among patients with neurological issues and score of 100% recognized as full confident and 0% indicated no confidence in performing activities [44].

### Statistical analysis

To enter data and analyze statistics, SPSS version 24 was used. A descriptive analysis was conducted using mean, median, mode, variance, and standard deviation for quantitative data like age, gender, age of onset of PD, and the diagnosis of PD. Kolmogorov–Smirnov and Shapiro–Wilk tests were used to determine the normality of the data. In both the control and experimental groups, data did not follow a normal distribution. To determine which intervention was effective, mean scores were evaluated. A p-value of less than 0.05 was considered statistically significant. The results of the analysis showed that the intervention was effective in reducing PD symptoms. In this study, the outcomes were compared between the three groups using one-way ANOVA and within the three groups using repeated measures ANOVA at a significance level of 0.05.

## Results

A total of 66 subjects were recruited and 60 that fulfilled the eligibility criteria were randomized to three groups; RPT with VR, RPT with MI and RPT. Baseline characteristics of the population studied showed that the mean ± standard deviation of age for VR & RPT group was 63.20 ± 4.85, for MI & RPT was 64.85 ± 5.10 and for RPT group was 61.95 ± 4.62. In case of gender the mean scores for females in VR & RPT group was 8, for MI & RPT group was 10 and for RPT group was 9. For males the mean values in VR & RPT group were 12, for MI & RPT group was 10 and for RPT group was 11. The mean values of height (cm) in VR & RPT group were 160.90 ± 3.07, in MI & RPT group was 162.85 ± 3.7 and for RPT group was 164.50 ± 2.66. In case of weight (kg) the mean values of VR & RPT group were 58.60 ± 4.97, in MI & RPT group was 62.60 ± 5.43 and for RPT group was 60.55 ± 4.96. For BMI the mean values of VR & RPT group were 22.68 ± 2.42, in MI & RPT group was 23.68 ± 2.53 and for RPT group was 22.42 ± 2.32. The mean score of Disease duration (years) in case of VR & RPT group was 6.65 ± 1.59, for MI & RPT group was 6.40 ± 2.50 and for RPT group was 6.25 ± 1.77. The mean age at onset of PD for RPT &VR group was 55.90 ± 4.16, for MI & RPT was 57.90 ± 5.48 and for RPT group was 56.10 ± 4.14. The mean age at diagnosis of PD for RPT &VR group was 59.80 ± 3.86, for MI & RPT was 61.05 ± 5.13 and for RPT group was 59.75 ± 3.90. The mean score of H&Y Stage for VR & RPT group was 2.07 ± 0.75, for MI & RPT group was 2.10 ± 0.61 and for RPT group was 2.32 ± 0.63. The mean score of MMSE in RPT &VR group was 26.50 ± 0.68, for RPT &MI was 26.40 ± 1.14 and for RPT was 26.65 ± 0.93. The mean Levodopa equivalent daily dose (LEDD) values for VR & RPT group was 455.00 ± 74.16, for MI & RPT group was 485.00 ± 102.72 and for control group was 465.00 ± 58.71 (Table 1).

**Table 1:** Demographics and clinical characteristics of the study subjects (*N* = 80)

Randomized (*n* = 60)				
Variables	Virtual reality (VR + RPT)	Motor imagery (MI + RPT)	Control Group (RPT alone)	
	(*n* = 20)	(*n* = 20)	(*n* = 20)	p-value
Age (years)	63.20 ± 4.85	64.85 ± 5.10	61.95 ± 4.62	0.762
Gender				0.817
Female	8	10	9	
Male	12	10	11	
Height (cm)	160.90 ± 3.07	162.85 ± 3.7	164.50 ± 2.66	0.217
Weight (kg)	58.60 ± 4.97	62.60 ± 5.43	60.55 ± 4.96	0.847
BMI	22.68 ± 2.42	23.68 ± 2.53	22.42 ± 2.32	0.13
Disease duration (years)	6.65 ± 1.59	6.40 ± 2.50	6.25 ± 1.77	0.907
Age at onset of PD	55.90 ± 4.16	57.90 ± 5.48	56.10 ± 4.14	0.978
Age at diagnosis PD	59.80 ± 3.86	61.05 ± 5.13	59.75 ± 3.90	0.893

PD; Parkinson’s disease, MMSE; Mini mental state examination, H&Y: Hoehn and Yahr Stage LEDD; levodopa equivalent daily dose

In case of activities of daily living, for UPDRS part-II the mean and standard deviation for VR & RPT group at baseline was 25.20 ± 3.036, at 6th week was 18.45 ± 3.590, at 12th week was 15.30 ± 2.364 and at 16th week the mean scores were 14.85 ± 2.814. The mean and standard deviation for MI & RPT group at baseline was 24.25 ± 3.522, at 6th week was 19.60 ± 3.844, at 12th week was 16.30 ± 2.848 and at 16th week the mean scores were 16.60 ± 4.210. The mean and standard deviation for RPT group at baseline was 24.85 ± 2.719, at 6th week was 21.55 ± 3.379, at 12th week was 19.50 ± 2.964 and at 16th week the mean scores were 18.70 ± 4.001. Thus, the VR & RPT group showed statistically significant results. **(**Table 2**)**

In case of motor function, for UPDRS part-III the mean and standard deviation for VR & RPT group at baseline was 33.95 ± 3.50, at 6th week was 24.00 ± 6.712, at 12th week was 17.20 ± 9.451 and at 16th week the mean scores were 18.15 ± 9.455. The mean and standard deviation for MI & RPT group at baseline was 32.70 ± 3.062, at 6th week was 25.20 ± 4.237, at 12th week was 18.90 ± 7.785 and at 16th week the mean scores were 19.90 ± 8.097. The mean and standard deviation for RPT group at baseline was 33.05 ± 3.136, at 6th week was 28.80 ± 4.572, at 12th week was 24.45 ± 5.266 and at 16th week the mean scores were 24.85 ± 4.738. Thus the VR & RPT group showed statistically significant results. (Table 2**)**

**Table 2:** Difference between groups regarding the mean scores of UPDRS-Part II & III

Outcome	Outcome Measures	Groups	Baseline	Assessment at 6th Week	Assessment at 12th Week	Follow up at 16th Week
			Mean ± SD	Mean ± SD	Mean ± SD	Mean ± SD
Motor Function	UPDRS-Part III	VR + RPT	33.95 ± 3.501	24.00 ± 6.712	17.20 ± 9.451	18.15 ± 9.455
		MI + RPT	32.70 ± 3.062	25.20 ± 4.237	18.90 ± 7.785	19.90 ± 8.097
		RPT	33.05 ± 3.136	28.80 ± 4.572	24.45 ± 5.266	24.85 ± 4.738
		F	0.793	4.462	4.854	4.084
		p-value	0.458	0.016	0.011	0.022
Activities of Daily livings	UPDRS-Part II	RPT + VR	25.20 ± 3.036	18.45 ± 3.590	15.30 ± 2.364	14.85 ± 2.814
		RPT + MI	24.25 ± 3.522	19.60 ± 3.844	16.30 ± 2.848	16.60 ± 4.210
		RPT	24.85 ± 2.719	21.55 ± 3.379	19.50 ± 2.964	18.70 ± 4.001
		F	0.477	3.770	12.839	5.352
		p-value	0.623	0.029	0.000	0.007

UPDRS; Unified Parkinson’s Disease Rating Scale

In case of balance, for BBS the mean and standard deviation for VR & RPT group at baseline was 37.15 ± 3.437, at 6th week was 44.75 ± 4.203, at 12th week was 50.10 ± 4.897 and at 16th week the mean scores were 51.65 ± 3.631. The mean and standard deviation for MI & RPT group at baseline was 37.90 ± 3.370, at 6th week was 42.85 ± 4.568, at 12th week was 47.45 ± 5.510 and at 16th week the mean scores were 47.40 ± 5.265. The mean and standard deviation for RPT group at baseline was 38.45 ± 4.322, at 6th week was 41.65 ± 3.759, at 12th week was 45.50 ± 4.559 and at 16th week the mean scores were 46.95 ± 4.058. Thus, the VR & RPT group showed statistically significant results. **(**Table 3**)**

In case of balance confidence, for ABCS the mean and standard deviation for VR & RPT group at baseline was 57.95 ± 4.629, at 6th week was 70.63 ± 3.636, at 12th week was 78.59 ± 6.386 and at 16th week the mean scores were 76.93 ± 6.705. The mean and standard deviation for MI & RPT group at baseline was 58.27 ± 8.341, at 6th week was 68.23 ± 6.442, at 12th week was 74.76 ± 6.467and at 16th week the mean scores were 71.23 ± 6.757. The mean and standard deviation for RPT group at baseline was 57.64 ± 8.767, at 6th week was 65.24 ± 8.091, at 12th week was 71.56 ± 8.090 and at 16th week the mean scores were 70.36 ± 9.027. Thus the VR & RPT group showed statistical improvement. **(**Table 3**)**

**Table 3:** Difference between groups regarding the mean scores of ABCS & BBS

Outcomes	Outcome Measures	Groups	Baseline	Assessment at 6th Week	Assessment at 12th Week	Follow up at 16th Week
			Mean ± SD	Mean ± SD	Mean ± SD	Mean ± SD
Balance confidence	ABCS	VR + RPT	57.95 ± 4.629	70.63 ± 3.636	78.59 ± 6.386	76.93 ± 6.705
		MI + RPT	58.27 ± 8.341	68.23 ± 6.442	74.76 ± 6.467	71.23 ± 6.757
		RPT	57.64 ± 8.767	65.24 ± 8.091	71.56 ± 8.090	70.36 ± 9.027
		F	0.036	3.641	5.014	4.441
		p-value	0.965	0.032	0.010	0.016
Balance	BBS	VR + RPT	37.15 ± 3.437	44.75 ± 4.203	50.10 ± 4.897	51.65 ± 3.631
		MI + RPT	37.90 ± 3.370	42.85 ± 4.568	47.45 ± 5.510	47.40 ± 5.265
		RPT	38.45 ± 4.322	41.65 ± 3.759	45.50 ± 4.559	46.95 ± 4.058
		F	0.610	2.783	4.256	7.032
		p-value	0.547	0.070	0.019	0.002

ABCS; Activities-specific Balance Confidence scale, BBS; Berg Balance Scale

Repeated measures ANOVA was used for within group analysis. For motor function the UPDRS Part III showed statistically significant results (*p* < 0.001) within RPT + VR group, RPT + MI and RPT group. The mean and standard deviation for VR & RPT group at baseline was 33.95 ± 3.50, at 6th week was 24.00 ± 6.712, at 12th week was 17.20 ± 9.451 and at 16th week the mean scores were 18.15 ± 9.455 with p-value less than 0.001. The mean and standard deviation for MI & RPT group at baseline was 32.70 ± 3.062, at 6th week was 25.20 ± 4.237, at 12th week was 18.90 ± 7.785 and at 16th week the mean scores were 19.90 ± 8.097 with p-value less than 0. 001. The mean and standard deviation for RPT group at baseline was 33.05 ± 3.136, at 6th week was 28.80 ± 4.572, at 12th week was 24.45 ± 5.266 and at 16th week the mean scores were 24.85 ± 4.738 with P-value less than 0.001. Thus, for motor function the UPDRS Part III showed statistically significant results (*p* < 0.001) within RPT + VR group, RPT + MI and RPT group. (Table 4**)**

For balance confidence the ABCS showed statistically significant results (*p* < 0.001) within RPT + VR group, RPT + MI and RPT group. The mean and standard deviation for VR & RPT group at baseline was 57.95 ± 4.629, at 6th week was 70.63 ± 3.636, at 12th week was 78.59 ± 6.386 and at 16th week the mean scores were 76.93 ± 6.705 with P-value less than 0.001. The mean and standard deviation for MI & RPT group at baseline was 58.27 ± 8.341, at 6th week was 68.23 ± 6.442, at 12th week was 74.76 ± 6.467and at 16th week the mean scores were 71.23 ± 6.757 with P-value less than 0.001. The mean and standard deviation for RPT group at baseline was 57.64 ± 8.767, at 6th week was 65.24 ± 8.091, at 12th week was 71.56 ± 8.090 and at 16th week the mean scores were 70.36 ± 9.027 with P-value less than 0.001. Thus, for balance confidence the ABCS showed statistically significant results (*p* < 0.001) within RPT + VR group, RPT + MI and RPT group. (Table 4**)**

For balance BBS showed statistically significant results (*p* < 0.001) within RPT + VR group, RPT + MI and RPT group. The mean and standard deviation for VR & RPT group at baseline was 37.15 ± 3.437, at 6th week was 44.75 ± 4.203, at 12th week was 50.10 ± 4.897 and at 16th week the mean scores were 51.65 ± 3.631 with p-value less than 0.001. The mean and standard deviation for MI & RPT group at baseline was 37.90 ± 3.370, at 6th week was 42.85 ± 4.568, at 12th week was 47.45 ± 5.510 and at 16th week the mean scores were 47.40 ± 5.265 with P-value less than 0.001. The mean and standard deviation for RPT group at baseline was 38.45 ± 4.322, at 6th week was 41.65 ± 3.759, at 12th week was 45.50 ± 4.559 and at 16th week the mean scores were 46.95 ± 4.058 with P-value less than 0.001. Thus, for balance BBS showed statistically significant results (*p* < 0.001) within RPT + VR group, RPT + MI and RPT group. (Table 4**)**

For Activities of daily living UPDRS Part II showed statistically significant results (*p* < 0.001) within RPT + VR group, RPT + MI and RPT group. The mean and standard deviation for VR & RPT group at baseline was 25.20 ± 3.036, at 6th week was 18.45 ± 3.590, at 12th week was 15.30 ± 2.364 and at 16th week the mean scores were 14.85 ± 2.814 with P-value less than 0.001. The mean and standard deviation for MI & RPT group at baseline was 24.25 ± 3.522, at 6th week was 19.60 ± 3.844, at 12th week was 16.30 ± 2.848 and at 16th week the mean scores were 16.60 ± 4.210 with P-value less than 0.001. The mean and standard deviation for RPT group at baseline was 24.85 ± 2.719, at 6th week was 21.55 ± 3.379, at 12th week was 19.50 ± 2.964 and at 16th week the mean scores were 18.70 ± 4.001 with P-value less than 0.001. Thus, for Activities of daily living UPDRS Part II showed statistically significant results (*p* < 0.001) within RPT + VR group, RPT + MI and RPT group. (Table 4**)**

**Table 4:** With-in group comparison of mean scores of outcome measures

Outcomes	Outcome Measures	Groups	Baseline	Assessment at 6th Week	Assessment at 12th Week	Follow up at 16th Week	Repeated Measure ANOVA
			Mean ± SD	Mean ± SD	Mean ± SD	Mean ± SD	p-value
Motor Function	UPDRS-Part III	VR + RPT	37.15 ± 3.437	24.00 ± 6.712	17.20 ± 9.451	18.15 ± 9.455	< 0.001
		MI + RPT	32.70 ± 3.062	25.20 ± 4.237	18.90 ± 7.785	19.90 ± 8.097	< 0.001
		RPT	33.05 ± 3.136	28.80 ± 4.572	24.45 ± 5.266	24.85 ± 4.738	< 0.001
Balance confidence	ABCS	VR + RPT	37.15 ± 3.437	70.63 ± 3.636	78.59 ± 6.386	76.93 ± 6.705	< 0.001
		MI + RPT	58.27 ± 8.341	68.23 ± 6.442	74.76 ± 6.467	71.23 ± 6.757	< 0.001
		RPT	57.64 ± 8.767	65.24 ± 8.091	71.56 ± 8.090	70.36 ± 9.027	< 0.001
Balance	BBS	VR + RPT	37.15 ± 3.437	44.75 ± 4.203	50.10 ± 4.897	51.65 ± 3.631	< 0.001
		MI + RPT	37.90 ± 3.370	42.85 ± 4.568	47.45 ± 5.510	47.40 ± 5.265	< 0.001
		RPT	38.45 ± 4.322	41.65 ± 3.759	45.50 ± 4.559	46.95 ± 4.058	< 0.001
Activities of Daily livings	UPDRS-Part II	VR + RPT	25.20 ± 3.036	18.45 ± 3.590	15.30 ± 2.364	14.85 ± 2.814	< 0.001
		MI + RPT	24.25 ± 3.522	19.60 ± 3.844	16.30 ± 2.848	16.60 ± 4.210	< 0.001
		RPT	24.85 ± 2.719	21.55 ± 3.379	19.50 ± 2.964	18.70 ± 4.001	< 0.001

UPDRS; Unified Parkinson’s Disease Rating Scale, ABCS; Activities-specific Balance Confidence scale, BBS; Berg Balance Scale

In case of sub components of motor functions, for UPDRS part-III in tremor the mean and standard deviation for VR & RPT group at baseline was 6.350 ± 1.039, at 12th week was 3.300 ± 1.301. The mean and standard deviation for MI & RPT group at baseline was 5.850 ± 1.308, at 12th week was 4.150 ± 1.565 and the mean and standard deviation for RPT group at baseline was 6.000 ± 1.521, at 12th week was 4.850 ± 1.565. Thus, the VR & RPT group showed statistically significant results (0.007). In case of sub components of motor functions, for UPDRS part-III in Rigidity the mean and standard deviation for VR & RPT group at baseline was 4.950 ± 0.604, at 12th week was 2.750 ± 1.251. The mean and standard deviation for MI & RPT group at baseline was 4.550 ± 0.998, at 12th week was 3.600 ± 1.391and the mean and standard deviation for RPT group at baseline was 4.650 ± 0.875, at 12th week was 4.050 ± 1.503. Thus, the VR & RPT group showed statistically significant results (0.015). In case of sub components of motor functions, for UPDRS part-III in Bradykinesia the mean and standard deviation for VR & RPT group at baseline was 1.950 ± 0.510, at 12th week was 0.950 ± 0.510. The mean and standard deviation for MI & RPT group at baseline was 1.850 ± 0.366, at 12th week was 1.550 ± 0.604 and the mean and standard deviation for RPT group at baseline was 1.950 ± 0.223, at 12th week was 1.350 ± 0.745. Thus, the VR & RPT group showed statistically significant results (0.012). **(**Table 5**)**

**Table 5:** Difference between groups regarding the mean scores of sub components of motor function (tremors, rigidity and bradykinesia)

Outcomes	Groups	Baseline	Assessment at 6th Week	Assessment at 12th Week	Follow up at 16th Week
		Mean ± SD	Mean ± SD	Mean ± SD	Mean ± SD
Tremors	VR + RPT	6.350 ± 1.039	4.600 ± 1.667	3.300 ± 1.301	4.450 ± 1.276
	MI + RPT	5.850 ± 1.308	4.800 ± 1.823	4.150 ± 1.565	4.500 ± 1.317
	RPT	6.000 ± 1.521	5.650 ± 1.565	4.850 ± 1.565	5.650 ± 1.460
	F	0.773	2.18	5.482	5.027
	p-value	0.466	0.122	0.007	0.01
Rigidity	VR + RPT	4.950 ± 0.604	3.700 ± 1.031	2.750 ± 1.251	2.850 ± 1.136
	MI + RPT	4.550 ± 0.998	4.300 ± 1.128	3.600 ± 1.391	3.700 ± 1.625
	RPT	4.650 ± 0.875	4.200 ± 1.005	4.050 ± 1.503	4.100 ± 1.803
	F	1.221	1.852	4.537	3.402
	p-value	0.302	0.166	0.015	0.04
Bradykinesia	VR + RPT	1.950 ± 0.510	1.700 ± 0.470	0.950 ± 0.510	1.050 ± 0.604
	MI + RPT	1.850 ± 0.366	1.750 ± 0.444	1.550 ± 0.604	1.550 ± 0.604
	RPT	1.950 ± 0.223	1.950 ± 0.223	1.350 ± 0.745	1.450 ± 0.686
	F	0.45	2.242	4.739	3.492
	p-value	0.64	0.116	0.012	0.037

PD patients according to Hoehn and Yahr stages at baseline in VR + RPT was 25% in stage I, 40% in stage II and 35% in stage III, in MI + RPT group 15% in stage I, 45% in stage II and 40% in stage III, in RPT group 15% in stage I, 35% in stage II and 50% in stage III with non-significant p-value (0.820). PD patients according to Hoehn and Yahr stages at 12th week in VR + RPT were 50% in stage I, 35% in stage II and 15% in stage III, in MI + RPT group 25% in stage I, 50% in stage II and 25% in stage III, in RPT group 20% in stage I, 35% in stage II and 45% in stage III with non-significant p-value (0.121). **(**Table 6**)**

**Table 6:** Percentage wise changes in PD stages at 6th, 12th and 16th week in three groups

		Treatment Groups						p-value
		VR & RPT		MI & RPT		RPT alone		
		N	%	N	%	N	%	
Hoehn and Yahr Stage at Baseline	Stage I	5	25.00%	3	15.00%	3	15.00%	0.82
	Stage II	8	40.00%	9	45.00%	7	35.00%	
	Stage III	7	35.00%	8	40.00%	10	50.00%	
Hoehn and Yahr Stage at 6th Week	Stage I	7	35.00%	4	20.00%	4	20.00%	0.472
	Stage II	8	40.00%	9	45.00%	6	30.00%	
	Stage III	5	25.00%	7	35.00%	10	50.00%	
Hoehn and Yahr Stage at 12th Week	Stage I	10	50.00%	5	25.00%	4	20.00%	0.121
	Stage II	7	35.00%	10	50.00%	7	35.00%	
	Stage III	3	15.00%	5	25.00%	9	45.00%	
Hoehn and Yahr Stage at 16th Week	Stage I	9	45.00%	4	20.00%	3	15.00%	0.098
	Stage II	8	40.00%	11	55.00%	8	40.00%	
	Stage III	3	15.00%	5	25.00%	9	45.00%	

## Discussion

Virtualization in the field of rehabilitation has emerged as a new technological advancement. Technology is playing its role in the assessment and treatment of the patients and research as well thereby opening new paths for the researchers for integrating the use of VR and MI in the rehabilitation plans [45]. Therefore, the exposure of the researchers, academicians and patents to these technologies is necessary to get familiar with the pros and cons of individual techniques. The research also plays its part in revealing the adverse effects, if present [46]. The literature has been, recently, filled up with research projects incorporating VR and MI, individually or in combination. Nonetheless, little is known about the comparative effects of VR with routine physical therapy, MI and routine physical therapy and routine physical therapy alone. To the best of the authors’ knowledge, no study has yet examined these three groups in one study to obtain a candid picture of which therapeutic regime is actually effective or creates sustained effects. Based on the customized protocol for each study group, the study was conducted [47]. The authors report the first randomized controlled trial in which combining virtual reality along with routine PT was compared with motor imagery combined with routine PT to improve and with routine PT alone. The current study found that the virtual reality group showed greater improvements in functional outcomes than the motor imagery group and routine PT alone. The results of the study showed that virtual reality is a promising tool for improving.

Using the novel technologies, the study is novel in its characteristics as well. The patients enrolled in the study showed improved performance in lieu of improved motor function, balance and balance confidence as a result of treatment. Considering the performance in individual groups, it can be clearly stated that RPT + VR groups excelled in comparison to the RPT + MI and RPT only group with the sustainability of the effects at follow-up. Statistically and clinically significant results were found after the data analysis. Looking closely on the results obtained from the study it is revealed that the motor function improved significantly in the VR + RPT group after 6 months of therapy, continuing to show the same results till the termination of treatment protocol and going well-beyond the discontinuation of therapy (after a month). The readings were obtained by using UPDRS (section -III) that is known to be a valid and reliable tool for PD and other neurological dysfunctions as well [48, 49].

Recently, it has been noted that many of the researches available in literature are systematic reviews. The reason might be to have concluding evidence about the most beneficial therapeutic regime and timing of the treatment along with other protocols. In recent times, a systematic review has provided substantial evidence that VR has beneficial effects for improvising the motor function and ADLS’s [50]. The cause of the effects created by the VR on the motor function might be the fact that VR may have helped patients in learning and acquiring new skills based on the repetitive actions, in-person engagement in the treatment protocol and recalling the memory again and again, multiple times during the session and then again in every exercise session. Keeping in mind that the VR protocol was built on these notions, it might give clue that that VR training has provided the external feedback at a pace required for the patient with PD to promote motor learning and improving the balance function. On a broader perspective, the results obtained in the VR + RPT are exceedingly greatly from the results obtained in other groups of the study.

Measuring the therapy efficacy is not an easy task during the interventional studies. For ensuring that the results are not by chance or assessment errors but the therapy is effective in its true spirit, clinically significant differences are important to identify. Minimal clinically important difference (MCID) criteria for each outcome measure are vital in this context. Looking in detail at each of the outcome measure, difference of 11 points of UPDRS-III, and 5 points on BBS is required to assure the results in its true sense, a condition that is fulfilled when results of the study are explored [51, 52]. Fall proclivity is evident in patients with PD presenting with balance disorders [53].

In the current study, it was hypothesized that VR combined with routine PT or MI combined with routine PT would significantly improve balance in patients with PD compared to patients in the control group. It also hypothesized that the improvement magnitude would be more significant than previous research. In addition, it was hypothesized that the improvement would persist through a one-month follow-up. The well-established protocol used in this study resulted in evident results in all study groups but more significantly in VR + RPT group where robust improvements were seen. In previous studies, improved balance and a lower fall risk were reported, however the improvements in balance were not as substantial as those reported in the current study [54–57].. Noteworthy fact is the sustainability of the effects after the cessation of treatment. This impact reveled in the present study needs applaud as few studies have mentioned such retained results and many didn’t even report fall risk [45, 58, 59]. This study followed an established protocol and required the subjects to attend 36 interventions over the course of the study. The minimum attendance rate for enrollment in this study was at least 33 sessions attended by each participant.

Few of the studies in the literature have reinforced the use of mental imagery for the comparable background as that of physical exercises but with ‘non tiring effect’ and no end movement but same neural processes, therefore one step superior to routine exercises. These mental practices of skilled movements or task-specific movements lead to better motor learning as stated by Moshref et al.in their study [60]. The present study also revealed such results as the effects in the MI + RPT group were superior to RPT only group but less than the VR + RPT group.

Prospectively, the recent literature supports the use of virtual environment either immersive or non-immersive, for the contextual experience of the real life environment, therefore, duplicating the results in the form of improved performance of ADLs [61, 62]. This novel technological advancement helps the patients with neurological impairments in better judgment of positional sense and spatial orientation. These two abilities are categorically related to performance of muscle activity with contribution from the visual and somatosensory systems as well [63, 64]. Moreover, technology helps in the movement repetition, enhanced feedback and as motivation booster. This type of rehabilitation is also known to target attention span and executive functions of the sufferers, thereby activating alternate neural pathways and supporting the concept of neural plasticity [65].

There are some limitations to this study that need to be taken into account in future studies. Only those with mild-to-moderate PD symptoms were included in this study and advanced stages of PD were not included. Moreover, phenotypic subtypes tremor dominant (TD) and postural instability-gait disturbance (PIGD) of PD were not reported in current study. There is a need to gather data on the cost-effectiveness of VR and MI training compared with routine PT to incorporate these technological advancements more often into physical therapy. There isn’t much literature available reporting the cost of VR equipment. It is recommended that clinicians incorporate the findings of this study into their routine practice, since VR combined with RPT or MI combined with RPT is more effective than RPT alone. In addition, VR balance training may offer clinicians an interesting alternative to home exercise prescription.

## Conclusion

The findings of the current study suggest that the combination of VR and routine PT appeared to be the most effective treatment method for improving balance, motor function, and daily activities in patients with PD as compared to MI and routine PT and routine PT alone.

## Data Availability

The data generated or analyzed during this study are presented in this article and for further enquiries can be directed to the corresponding author.
